# The Chemistry of Stress: Understanding the ‘Cry for Help’ of Plant Roots

**DOI:** 10.3390/metabo11060357

**Published:** 2021-06-02

**Authors:** Muhammad Syamsu Rizaludin, Nejc Stopnisek, Jos M. Raaijmakers, Paolina Garbeva

**Affiliations:** 1 Department of Microbial Ecology, Netherlands Institute of Ecology (NIOO-KNAW), 6708 PB Wageningen, The Netherlands; N.Stopnisek@nioo.knaw.nl (N.S.); j.raaijmakers@nioo.knaw.nl (J.M.R.); 2 Institute of Biology, Leiden University, 2333 BE Leiden, The Netherlands; 3 Department of Plant and Environmental Sciences, Faculty of Natural and Life Sciences, University of Copenhagen, 1871 Copenhagen, Denmark

**Keywords:** abiotic and biotic stresses, cry-for-help, root exudates, volatiles, plant-microbe interactions

## Abstract

Plants are faced with various biotic and abiotic stresses during their life cycle. To withstand these stresses, plants have evolved adaptive strategies including the production of a wide array of primary and secondary metabolites. Some of these metabolites can have direct defensive effects, while others act as chemical cues attracting beneficial (micro)organisms for protection. Similar to aboveground plant tissues, plant roots also appear to have evolved “a cry for help” response upon exposure to stress, leading to the recruitment of beneficial microorganisms to help minimize the damage caused by the stress. Furthermore, emerging evidence indicates that microbial recruitment to the plant roots is, at least in part, mediated by quantitative and/or qualitative changes in root exudate composition. Both volatile and water-soluble compounds have been implicated as important signals for the recruitment and activation of beneficial root-associated microbes. Here we provide an overview of our current understanding of belowground chemical communication, particularly how stressed plants shape its protective root microbiome.

## 1. Introduction

Over the last decade, studies on plant metabolites have increased significantly due to substantial technological advances in platforms for metabolomic analyses. These studies have revealed new exciting insights into the chemical diversity of plant metabolites. Plants synthesize more than 200,000 primary and secondary metabolites, including volatile and soluble compounds [1]. While primary metabolism mainly involves compounds important for plant growth, development, and reproduction, specialized metabolism encompasses compounds needed to successfully cope with fluctuating abiotic and biotic stresses. The great diversity of secondary metabolites in plants stems from a limited number of building blocks. These scaffolds are ubiquitous in the majority of plants but differ in a species-specific manner, in enzymatic permutation, and in decoration of their basic structures. For example, one of the most highly diverse and biologically intriguing group of plant secondary metabolites is the terpenes, whose the biosynthesis is governed by terpene synthase genes that can generate volatile, semi-volatile and non-volatile derivatives [2]. A single terpene synthase gene can generate many different terpenes depending on the linear precursor: geranyl diphosphate (GPP, C_10_ monoterpenes), farnesyl diphosphate (FPP, C_15_ sesquiterpenes, and C_30_ triterpenes) and geranylgeranyl diphosphate (GGPP, C_20_ diterpenes, and C_40_ tetraterpenes) [3]. Plant secondary metabolites not only have important physiological functions but also a significant impact on plant ecology. By producing particular secondary metabolites, plants can provide detailed information about their physiological state. They can also in such way influence and manipulate the physiology of neighboring plants and the behaviour of other (micro)organisms [4,5,6,7]. For example, plant terpenes such as 1,8-cineole, (*E*)-β-ocimene, linalool, (*E*)-β-caryophyllene all play important roles in plant-insect, plant-pathogen and plant-plant interactions [8,9,10].

In their natural environment, plants are often exposed to a variety of biotic and abiotic stresses such as drought, salinity, nutrient limitations, pests and pathogens. To withstand these different stresses, plants have developed sophisticated adaptive mechanisms including the production of bioactive secondary metabolites. Some of these metabolites can have direct defensive effects while others can warn neighboring plants to mount their own defenses or lead to the recruitment of beneficial (micro)organisms that minimize the intensity of plant stresses both above-and belowground. It is well-known that, in response to attacks by aboveground herbivores, plants have evolved a ”cry-for-help” strategy where they recruit beneficial organisms to help to overcome the imposed stresses [11]. For example, the enhanced emission of terpenes results in attraction of the natural enemies of herbivores [12]. In most cases, the function of plant metabolites is best studied for aboveground plant responses including defense against biotic and abiotic stresses, as signaling molecules attracting pollinating insects and as plant phytohormones [13].

Recent studies have indicated that plants roots have also evolved a “cry-for-help” strategy to recruit beneficial soil (micro)organisms to minimize the damage caused by these stresses [14,15,16,17,18]. As beneficial soil microbes can help plants to overcome different stresses and improve plant growth, it is crucial for plants to recruit, activate and assemble protective microbiomes. However, studying plant metabolites produced belowground is a challenging task as soils are complex consisting of a heterogeneous matrix of water- and air-filled pores that are recalcitrant to chemical analysis [19,20]. Nevertheless, chemical communication is likely the most prevalent means in belowground interaction.

Increasing our fundamental knowledge of belowground chemical interactions can provide a basis for developing new strategies for the sustainable crop production. This review provides an overview of belowground, stress-induced chemical communication. We give a summary of the various soluble and volatile metabolites released by plants roots exposed to biotic and abiotic stresses and discuss their role in assembly of a plant protective microbiome and stress alleviation. Furthermore, we discuss the challenges, opportunities and future directions in this emerging research field.

## 2. Root Exudate Chemistry of Plants Exposed to Abiotic and Biotic Stresses

Plants release a significant fraction of their photosynthetically fixed carbon belowground in the form of root exudates [21], consisting of a diverse array of volatile and non-volatile compounds [22,23]. Shaped through a long evolutionary process, the exudation of root metabolites is among plants’ sophisticated strategies to survive in changing environments. External stress stimuli such as pathogen and pest attacks, heavy metal contamination, as well as nutrient and/or water limitation can lead to modification of carbon allocation belowground. Both the quantity and composition of root exudates can change upon plant exposure to different external stress factors [15,24]. Intriguingly, such stress-driven alteration in exudate profiles can directly enhance plants’ survival. For instance, increased secretion of organic acids, particularly malate has been observed in soybean plants grown under phosphorus (P) starvation [25,26]. Similarly, the response of roots of white lupine (*Lupinus albus* L.) to soil P deprivation induces the production of carboxylate [27,28]. Organic acids in the group of di-and tricarboxylic acids such as malate, carboxylate, and oxalate can replace inorganic phosphate (Pi) bound in insoluble P-metal complexes either via metal ion chelation or by anion exchange, leading to P mobilization and increased P uptake by roots [29,30,31].

Exudation of low molecular-weight organic acids (LMWOA) into the rhizosphere has also been reported as a survival strategy of plants to improve nutrient acquisition in metal-contaminated soils. For example, in acidic soils with high aluminum (Al) concentrations, roots of *Glycine max* (soybean) and *Zea mays* (maize) secrete citrate with a strong metal chelating capability, thereby reducing the uptake of harmful Al^3+^ by the plants [26]. Furthermore, under moderate drought conditions, roots can produce mucilage, a polysaccharide acting as lubricant to facilitate root movement through dry soils [32]. In addition to this, increased levels of the osmolytes proline and pinitol were found in roots of soybean exposed to drought [33]. These compounds can maintain cell turgor via active osmoregulation, thereby increasing plants’ survival amidst water scarcity [33].

Another class of compounds secreted by plants in response to different stresses are the phenolics. For instance, roots of *Arabidopsis thaliana* grown in iron (Fe)-deficient soils (due to high pH) produce the coumarin scopoletin that reduces Fe^3+^ to Fe^2+^, thereby enhancing Fe-bioavailability to plants upon alkaline stress [34,35]. Furthermore, the growth of *Arabidopsis* mutants lacking 2-oxoglutarate-dependent dioxygenase Feruloyl-CoA 69-hydroxylase1 (F6′H1), an enzyme involved in scopoletin biosynthesis, was significantly impaired in growth on synthetic agar media supplemented with low Fe [36]. Increased secretion of phenolic compounds was also shown for roots of barley plants (*Hordeum vulgare*) infected with the soil-borne pathogenic fungus *Fusarium graminearum* [14]. Among these compounds was the antifungal *t*-cinnamic acid [14]. Similarly, the antifungal rosmarinic acid was secreted by roots of sweet basil (*Ocimum basilicum*) upon infection by the oomycete pathogen *Pythium ultimum* [15].

Increased accumulation of the non-volatile terpenoids zealexins and kauralexins was found for roots of maize following infection of soil-borne fungus *Fusarium verticillioides* and the belowground herbivore *Diabrotica balteata* [37]. Interestingly, the study also showed that plants deficient in kauralexins production (*an2* mutant) were more sensitive to drought. These results suggest that the terpenoids, apart from their role in direct defense against pests and pathogens, are associated with drought tolerance in maize plants. In another study, the semi-volatile diterpene rhizathalene was produced by *Arabidopsis* roots exposed to the root-feeding insect (*Bradysia*) [38]. Furthermore, the volatile monoterpene 1,8-cineole and the sesquiterpene (*Z*)-γ-bisabolene have been reported in root exudates of *Arabidopsis* [39], both with antimicrobial effects [40]. Similarly, the monoterpene (*S*)-limonene appears to be involved in the direct defense against plant pathogenic fungus *Magnaporthe oryzae* [41].

**Figure 1:**
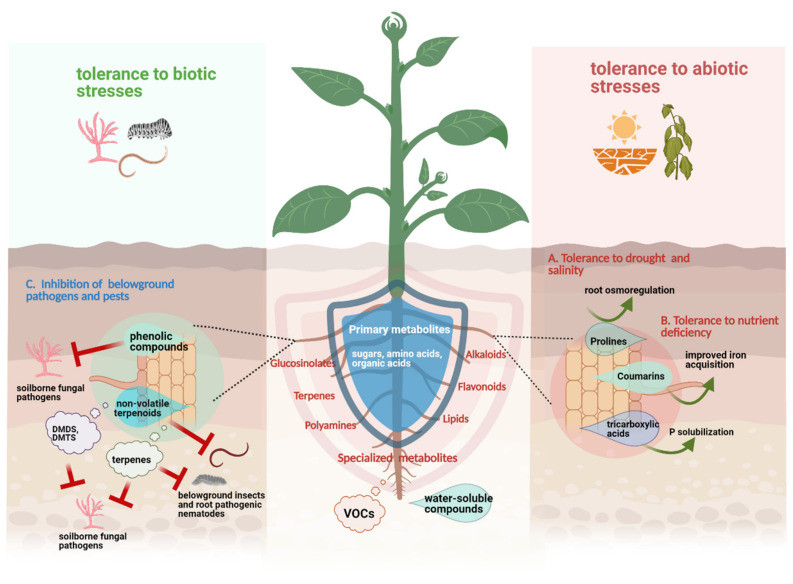
Schematic representation of the direct role of root exudates in plant resistance against biotic and abiotic stresses; drought and salinity (A), nutrient deficiency (B), and belowground pathogens and pests (C). Some specialized metabolites such as prolines, coumarins and organic acids can promote plant growth under abiotic stress conditions (i.e., drought, salinity, and nutrient deficiency) either via improved nutrient/mineral acquisition or active root osmoregulation. Meanwhile, upon a particular biotic stress, specialized root exudates such as phenolic compounds, non-volatile terpenoids, volatile terpenes and sulfurous compounds (i.e., dimethyl disulfide (DMDS), dimethyl trisulfide (DMTS)) are released and can directly inhibit the growth of invading soil-borne pathogens and pests. This figure was designed with Biorender (https://www.biorender.com, accessed on 18 April 2021).

Collectively, these findings indicate that the same compound classes can be released by roots of a particular plant species exposed to different stresses, suggesting a general role of some exudate constituents in mediating plant resistance against both biotic and abiotic stresses. The schematic overview on the direct role of root exudates on plant resistance against biotic and abiotic stresses can be seen in Figure 1.

## 3. Chemistry of Microbial Recruitment by Roots of Plants under Siege

Root-associated microorganisms are essential for plant growth and health. Past and present plant microbiome studies have indicated that root microbiota are not merely passengers, but instead, they improve plant immune functions [42] and enhance plant resilience to biotic and abiotic stresses [43]. In this context, it has been postulated that upon pathogen or pest attacks, plants change their root chemistry to actively recruit beneficial microbiota to facilitate adaptation and/or protection to the stresses, a phenomenon referred to as “cry for help” [14]. This phenomenon has been elegantly depicted in an early study where the infection of *Arabidopsis* leaves by *Pseudomonas syringae* pv *tomato* (*Pst*) attracted the beneficial bacterium *Bacillus subtilis* FB17 to the roots, that in turn triggered systemic resistance against subsequent infections by *Pst* [44]. Similarly, infection of *Arabidopsis* leaves by the downey mildew pathogen *Hyaloperonospora arabidopsidis* resulted in the enrichment of specific bacterial genera in the rhizosphere, in particular *Microbacterium* sp., *Stenotrophomonas* sp., and *Xanthomonas* sp. [45]. When applied to the soil individually or in mixture, these microbes were able to significantly reduce mildew incidence via induced systemic resistance (ISR) [45]. In a recent study, the belowground “cry for help” concept was also supported by results from a field experiment, where durum wheat (*Triticum turgidum* L. var *durum*) naturally infected by the crown-rot pathogen *Fusarium graminearum* enriched for *Stenotrophomonas rhizophila* (SR80) in the rhizosphere and root endosphere [16]. Upon re-introduction, strain SR80 was able to induce resistance against the crown-rot disease and enhance wheat growth [16].

Evidence is mounting that the recruitment of beneficial microbiota by plants under siege is, at least in part, driven by changes in the exudate profiles. These studies include the early work on malic acid secreted by *Arabidopsis* roots upon *Pst* infection [44]. This was further supported in the follow-up study showing that *Pst* infection enhanced the expression of Aluminum-Activated Malate Transporter1 (MLT1) which became the key regulator for the recruitment of *Bacillus subtilis* FB17 on *Arabidopsis* roots following foliar *Pst* infection [46]. In another study, local infection of cucumber roots by *Fusarium oxysporum* f.sp. *cucumerinum* increased tryptophan but reduced raffinose exudation; these changes enhanced root colonization by the beneficial bacterium *Bacillus amyloliquefaciens* SQR9 [47]. Furthermore, increased exudation of the fatty acid oxylipin was shown in tomato roots exposed to various stresses (wounding, salt, pathogen attack), in which the compound acted as a chemoattractant for the biocontrol fungus *Trichoderma harzianum* [48]. Benzoxazinoids, a class of defensive secondary metabolites commonly released by maize roots upon herbivory attacks can recruit the beneficial bacterium *Pseudomonas putida* KT2240 [49]. In the follow-up study, the same bacteria was shown to trigger induced systemic resistance (ISR) against *Colletotrichum graminicola* [50]. In a recent study, benzoxazinoids produced by maize plants was also found to alter the composition of root-associated microbiota, that in turn enhanced defense of leaves against the aboveground insect *Spodoptera frugiperda* [51].

While the concept of root exudate-mediated “cry-for-help” is shown for plants under biotic stresses, several studies suggest that this concept may also apply to plants exposed to abiotic stresses. For instance, roots of red clover (*Trifolium pratense*) grown in Fe-deficient soil accumulated phenolic compounds, which stimulated a siderophore-secreting *Pseudomonas* sp. When tested in plants, the siderophores produced by the bacterium were effective in solubilizing Fe, thereby improving its uptake by clover plants in Fe-deficient soils [52]. Furthermore, *Arabidopsis* roots are known to produce the coumarin scopoletin under Fe-deficiency [36]. The production of this compound was found to selectively impact the assembly of microbial community in the rhizosphere, resulting in enhanced plant growth under Fe limitation [53]. Under salt stress, roots of the halophyte *Limonum sinense* secreted several organic acids including 2-methyl butyric acid and palmitic acid with positive effects on the growth and chemotaxis of *Bacillus flexus* KLBPM 491, a beneficial bacterium naturally found on *L. sinense*. Re-introduction of this strain into soil significantly promoted growth of *L. sinense* seedlings under salinity stress [54]. Hence, root-derived exudates, apart from their direct role in plant defense, may also attract and activate plant-protective members of the rhizosphere microbiome to alleviate the imposed stresses. The summary of representative studies on direct and indirect role of root exudates on plant fitness can be seen in Table 1.

## 4. Chemistry of “Volatile Affairs” on Plant Roots

Apart from soluble compounds, roots release various volatile organic compounds (VOCs) into the rhizosphere. It is estimated that VOCs account for approximately 1% of the total secondary metabolites released by roots [61]. Plant VOCs are mainly represented by terpenoids, phenylpropanoids/benzenoids, fatty acid and amino acid derivatives [9]. Although VOCs are considered as minor component of root exudates, root VOCs hold a significant role in plant stress resilience. In general, VOCs have low molecular weight, with a lipophilic character and low boiling points [62]. Due to these physicochemical properties, root-emitted VOCs can easily diffuse via both air- and water filled pores in the soil and therefore, can cover long-distance chemical interactions. In the last years, the realization that VOCs play integral part in the belowground interactions has increased research attention in analysis of belowground VOCs [63].

Similar to soluble compounds, belowground plant VOCs can serve as direct and indirect plant defenses. For example, VOCs emitted from glucosinolate or cyanogenic glycoside conversion (such as cyanides and isothiocyanates) may serve as direct plant defenses as they are toxic to a wide range of belowground herbivories and pathogens [64,65]. Plant root VOCs can play important role as indirect plant defenses, e.g., attracting natural enemies or predators. One of the first studies on the indirect defenses via VOCs function belowground revealed that entomopathogenic nematodes were attracted to the roots of *Thuja occidentalis* damaged by larvae of the black vine weevil (*Otiorhynchus sulcatus*) when given a choice in a Y-tube olfactometer filled with sand [66]. In another study, maize roots damaged by the rootworm *Diabrotica virgifera virgifera* emit the sesquiterpene (E)-β-caryophyllene which attracts entomopathogenic nematodes that infest and kill the root-feeding larvae [67].

Root VOCs can act as both signaling molecules and nutrient sources for soil microbes [68], and hence might influence the assembly and proliferation of root-associated microbiome. A microcosm experiment showed that VOC-derived carbon released (in the headspace) during decomposition of ^13^C-labelled leaf litter accounted for fractions in microbial biomass (located separately from the decomposition site), suggesting that VOCs can be carbon sources for soil microbes [69]. In another study, gaseous ethylene produced by peanut roots (as a response to cyanide released by neighboring cassava plants) altered microbial composition of peanut rhizosphere by shifting the abundance of actinobacterial species, resulting in improved seed production [60]. This finding indicates that plants response to an environmental stimulus (cyanide-derived signal) via production of root-VOCs leading to the assembly of rhizosphere microbial community.

Evidence is available that under a biotic stress situation, root VOCs are involved in the recruitment of beneficial bacteria into the rhizosphere. This phenomenon had been clearly shown in a recent study by Schulz-Bohm et al. [58] who found that upon the infection by *Fusarium culmorum*, the root of sand sedge plant (*Carex arenaria*) emitted a specific blend of VOCs (i.e., including the monoterpene (*Z*)-limonene oxide) which clearly differed from the healthy plants. Interestingly, this specific blend of VOCs was able to attract certain beneficial bacteria within a synthetic bacterial community from a distance of approximately 12 cm. Furthermore, these recruited bacteria were able to inhibit the growth of *F. culmorum* [58].

Recently, a unique olfactometer-choice assay was applied to assess the migration of selected beneficial bacteria towards the roots of healthy and infected tomato plants in a soil system [59]. The study revealed that the infection of tomato plants with the fungal pathogen *Fusarium oxysporum* alters the root VOCs profile. The infected plant roots emitted VOCs such as benzonitrile, benzothiazole, dimethyl trisulfide, formic acid and a terpene-like compound, which are well-known for their antifungal activities. Interestingly, the infected and healthy plant roots did not show significant difference in the attraction of bacteria with biocontrol properties. However, these results were obtained only based on few selected bacteria; therefore, follow-up studies should be performed using total microbial communities to reveal whether the attraction of bacteria is significantly different between healthy and infected plant roots. Together, these findings suggest a novel strategy by which stressed plants can recruit and activate from distance soil microbes into the rhizosphere facilitating their adaptation. However, the role and mechanisms of root VOCs in the assemble and function of rhizosphere microbiome remain largely unexplored.

Interestingly, both plant-beneficial and plant-pathogenic microbes can modify the plant VOCs profile. For example, the pretreatment of faba bean (*Vicia faba* L.) plants with symbiotic arbuscular mycorrhiza fungi (AMF) reduced the release of sesquiterpenes [70]. The AMF suppressed emission of the sesquiterpenes (*E*)-caryophyllene and (*E*)-β-farnesene, and aphid attractiveness to VOCs was negatively associated with the proportion of sesquiterpenes in the sample. Hence, the AMF have a key bottom-up role in insect host location by increasing the attractiveness of aphids to plant VOCs. Recent study revealed that the plant-beneficial *Pseudomonas putida* induced the production of indole and β-caryophyllene VOCs in maize plants and triggered ISR against the maize anthracnose fungus *Colletotrichum graminicola* [50].

Furthermore, microbe induced plant VOCs can interfere with plant-to-plant communication. Recently, the effect of volatiles on microbial communities and neighboring plants was investigated in tomato plants (*S*. *lycopersicum* L.) inoculated with plant growth-promoting rhizobacterium *Bacillus amyloliquefaciens* [71]. The study revealed that tomato plants treated with bacteria released β-caryophyllene, which elicited the release of a large amount of salicylic acid in the root exudates of neighboring tomato plants affecting their rhizosphere microbiome. Hence, plants are able via microbe-induced plant VOC production to effect the rhizosphere microbiome of neighboring plants from a distance.

Considering the importance of root-emitted VOCs (next to the water-soluble exudates) on the assembly of protective-microbiome under stress conditions, future studies should elucidate more on the specificity or generality of root-emitted VOCs under different stresses, and investigate whether such volatile emissions can mediate the recruitment of beneficial microbes and activation of their beneficial traits.

## 5. Challenges, Opportunities, and Future Directions

New developments in metabolomics offer a great potential to gain new insights into the mechanisms underlying stress-induced belowground chemical interactions as it allows for the discovery of novel compounds or combinations of compounds that directly or indirectly alleviate (a) biotic stresses. In general, detection of plant metabolites is extremely challenging, as there is no single-instrument platform available to effectively measure the overall coverage of plant metabolites and standards for many root exudates are currently lacking. To date, mass spectrometry-based metabolomic techniques (such as LC-MS and GC-MS) are the most sensitive for simultaneous analysis of a large number of soluble and volatile compounds [72]. Especially mass spectrometry imaging (MSI) has emerged as a valuable tool, with numerous applications in the field of plant metabolomics [73]. MSI was used for spatio-temporal metabolite and elemental profiling of salt stressed barley during initial stages of plant germination [74]. MSI analytical techniques enable high-resolution spatial mapping of a large variety of biomolecules, providing qualitative and quantitative chemical information, in a single experiment. MSI can help to spatially elucidate the metabolite composition of the intact roots with minimal to no sample preparation [75]. Yet, the precise identification of the relevant plant metabolites and the assessment of their exact function remain a difficult and time-consuming process. In contrast to genes and proteins, metabolites have much greater structural diversity: they are not simply combinations of 4–20 letters of the gene or protein alphabet. The developments and combinations of novel metabolomics approaches and bioinformatics pipelines to search multiple databases for the identification of compounds in a metabolomics profile are crucial and urgently needed (e.g., [76,77]).

Most studies, so far, are focused on plant metabolites induced by individual stress under controlled conditions, but in nature, plants are typically subjected to a multiple stress factors at the same time. To date, little is known about plant metabolic responses to multiple stresses that occur either simultaneously or sequentially. Although several stresses have similarities (i.e., damaging plant tissue), each stress can lead to specific metabolic responses. Furthermore, the chemical responses to stress can depend on stress severity and duration [78]. For example, mild and short time aboveground stress seldom elicits release of stress VOCs, whereas severe stresses lead to major qualitative and quantitative changes in VOCs emission [79].

While responses to stresses are studied predominantly with young plants very little is learned about how mature plants perceive stresses and recruit or activate beneficial members of the root microbiome through chemical cues. Root exudates have been shown to change significantly during plant development and having an impact on microbiome composition [80]. In the case of 18 grass species, drought had a conserved effect on the plant microbiome in younger plants while microbiome of the drought stressed mature plants were more species-specific yet diverse members of Actinobacteria were enriched in both cases [81]. Whether these observations relate to different qualities and/or quantities of plant exudates or due to structural changes related to plant development remains unknown.

The majority of studies on stress-induced plant metabolites are based on experiments with domesticated plants and hence, we know very little about the responses of the wild relatives in natural ecosystems. Both in agricultural and natural ecosystems, plants are subject to multiple biotic and abiotic stresses. Even if they are resilient to a single stress, plants are often compromised to tolerate multiple stresses occurring at the same time. It is plausible that plants under multiple stresses produce different chemical cues, resulting in the attraction of different microbes compared to plants under single stress stimuli. Interactive effects of biotic and abiotic stress responses are reflected in the plant’s hormonal signaling networks. Crosstalk in this signaling network regulates secondary metabolite biosynthesis and the metabolites produced by the plant may serve as cues for soil microbes that may mitigate the adverse effects.

**Figure 2:**
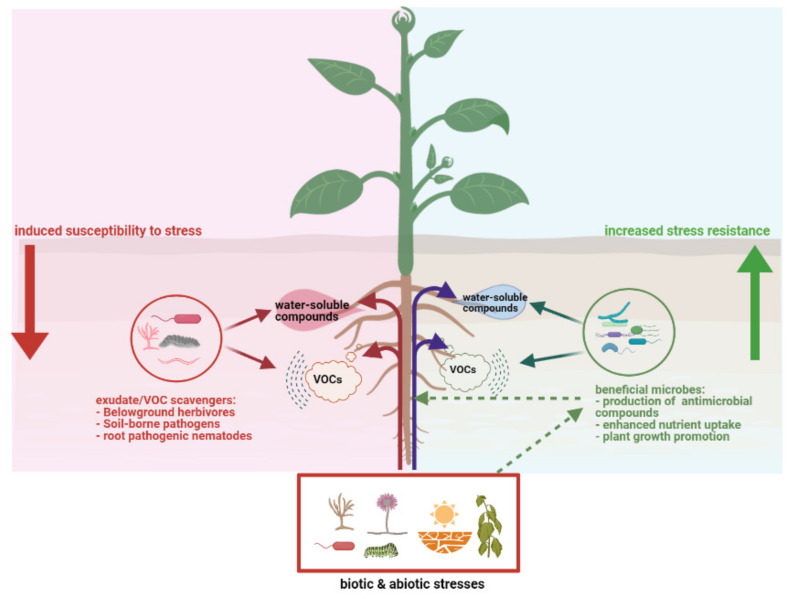
Schematic overview of how plants under siege can attract via exudation of specialized volatile and water-soluble root exudates, beneficial microorganisms which in turn can enhance plant fitness (**right panel**). At the same time, the exudates can also be used by scavengers such as belowground pathogens and pests as chemical information to locate and benefit from the stressed plants, leading to induced susceptibility to stresses (**left panel**). Furthermore, the biotic and abiotic stresses such as drought, salinity, nutrient limitations and pathogens, pests are stress factors not only for the plants but also for the root-associated microbiome (right panel) which can provide early warning and protection to the plant. This figure was designed with Biorender (https://www.biorender.com, accessed on 18 April 2021).

Apparently plant roots have evolved a “cry-for-help” strategy whereby they recruit beneficial soil microorganisms that can help to overcome stresses (Figure 2). However, information is required on the chemistry, dynamics, and mechanisms underlying the stress-induced recruitment as well as on the functional traits and genes of the recruited microbes. Such knowledge is currently lacking. In addition, a knowledge gap exists regarding the extent to which “cry-for-help” strategy is applicable across different types of stresses. Stress-emitted plant metabolites may provide information not only to beneficial microbes that help to alleviate stress, but to plant pathogens or scavengers as well, which might profit from the stressed plant (Figure 2).

Furthermore, several metabolites (e.g., indole, sulphur compounds, terpenoids) are commonly produced by both plant roots and plant associated organisms. The biotic and abiotic stresses such as drought, salinity, nutrient limitations, and pathogens are stress factors not only perceived by plants but also plant microbiome. Hence it is intriguing to know who is the first to sense the stress; is it the plant or plant associated microbes? Previous study revealed that the rhizosphere bacteria of the species *Serratia plymuthica* upregulated their terpene synthase gene and produced sodorifen together with as yet structurally unknown terpenes as a response to terpenes produced by the fungal root pathogen *Fusarium culmorum* [82]. Further experiments revealed that exposure of *Arabidopsis* seedlings to the *Serratia* produced sodorifen induced expression of two plant defense-related genes PDF1.2 and PR1, coinciding with reduced infections by the pathogen [83]. Hence, it is plausible, that plant associated microbes are the first to sense the stress and produce specific metabolites to alert their host plant.

Climate change is expected to lead to increased frequency and severity of drought and rainfall events in the near future. These extreme events will have a strong impact on the belowground chemical communication, as water is the major medium for moving molecules into the soil space. Under such condition, volatile compounds can play even more important role in belowground chemical interactions as they can easily diffuse in the gas phase which is not possible for soluble compounds. In addition to abiotic stresses, plants are constantly facing attacks by pests and pathogens which are also expected to increase due to climate change. Plant-associated microbes can alleviate both biotic and abiotic stresses through mechanisms as diverse as induction of plant resistance, direct antagonism of pathogens, increased nutrient availability, or modulation of plants’ hormonal balance. The rhizosphere microbiome thus is a reservoir of efficient helpers that plants can specifically recruit to help them cope with one or multiple stresses. Therefore, deciphering belowground chemical communication can provide a fundamental knowledge for developing the multiple-stress resistant crops in the future.

## Figures and Tables

**Table 1:** Representative studies where plants under various stresses can produce specific root metabolites that can directly and indirectly (via recruitment/modulation of beneficial root associated microbiome) affect plant fitness.

Stress Type	Plant Species	Type of Exudation	Role of Exudate in Plant Defence	Reference
**Biotic stress**				
*Fusarium graminearum*	Barley *(Hordeum vulgare)*	*t*-Cinnamic acid (water-soluble)	Direct via antifungal activity	[55]
*Pythium ultimum*	Sweet Basil *(Ocimum bacilicum)*	Rosamarinic acid (water-soluble)	Direct via antifungal activity	[56]
*Fusarium verticillioides; Diabrotica balteata*	Maize (*Zea mays*)	Terpenoids; zealexins, kauralexins	Direct via antifungal activity and suppression of herbivory growth	[37]
*Bradysia spp.*	*Arabidopsis thaliana*	Rhizathalene (semi volatile)	Direct via suppression of herbivory growth	[38]
*Pseudomonas syringae* pv *tomato*	*Arabidopsis thaliana*	L-Malic acid (water-soluble)	Indirect via recruitment of *Bacillus subtilis* F017	[44]
*Pythium ultimum*	Barley *(Hordeum vulgare var. Barke)*	Phenolic compounds (water-soluble)	Indirect via activation of phlA genes (required for antifungal production) of *Pseudomonas* *fluorescens*	[57]
*Fusarium oxysporum* f.sp. *cucumerinum*	Cucumber *(Cucumis sativus)*	Tryptophan (water- soluble)	Indirect via increased colonization of plant growth promoting rhizobacterium (PGPR) *Bacillus* *Amyloliquefaciens* SQR9	[47]
*Fusarium culmorum*	Carex (*Carex arenaria*)	Monoterpene (*Z*)- limonene-oxide (volatile organic compound)	Indirect via attraction of *Janthinoacterium*, *Collimonas*, and *Paenibacillus* showing antifungal activities	[58]
*Fusarium oxysporum*	Tomato (*Solanum lycopersicum* cv. Hildares	Benzonitrile, benzothiazole, dimethyl trisulfide, formic acid and a terpene-like compound (volatile organic compounds)	Direct via antifungal activities; indirect via attraction of Bacillus spp.	[59]
Cassava (*Manihot esculenta*), neighboring plants	Peanut (*Arachis hypogaea* L.)	Ethylene (volatile organic compounds)	Indirect via increase the abundance of an Actinobaterial species (Catenulispora) able to enhance seed production	[60]
**Abiotic stress**				
P starvation	White Lupine *(Lupinus albus*	Carboxylate (water- soluble)	Direct via phosphate solubilization	[31]
P starvation	Soybean (*Glycine max*)	Malate (water- soluble)	Direct via phosphate solubilization	[25]
Drought	Soybean (*Glycine max*)	Proline; pinitol (water-soluble)	Direct via active osmoregulation	[33]
Aluminium toxicity	Maize *(Zea mays;* soybean *(Glycine max)*	Citrate	Direct via metal chelation limiting Al uptake	[26]
Iron deficiency	Red clover *(Trifolium pratense)*	Phenolic compounds	Indirect via recruitment of bacterial community able to produce siderophore in the rhizosphere	[52]
Iron deficency	*Arabidopsis thaliana*	Coumarin scopoletin	Indirect via recruitment of several bacterial genera having plant growth promoting properties	[53]
Salinity stress	Halophyte (*Limonum sinense*)	2-Methylbutyric acid and palmitic acid	Indirect via recruitment of *Bacillus flexus* KLBPM 491 able to enhance plant growth under salinity stress	[54]
